# A Comparison of Gene Expression Changes in the Blood of Individuals Consuming Diets Supplemented with Olives, Nuts or Long-Chain Omega-3 Fatty Acids

**DOI:** 10.3390/nu12123765

**Published:** 2020-12-08

**Authors:** Virginie Bottero, Judith A. Potashkin

**Affiliations:** Center for Neurodegenerative Disease and Therapeutics, The Chicago Medical School, Rosalind Franklin University of Medicine and Science, North Chicago, IL 60064, USA; virginie.bottero@rosalindfranklin.edu

**Keywords:** nutrigenomic, olive, nut, fish, Mediterranean diet, nuclear factor interleukin 3 regulated, *NFIL3*, dementia, Alzheimer’s disease

## Abstract

Background: The Mediterranean diet, which is rich in olive oil, nuts, and fish, is considered healthy and may reduce the risk of chronic diseases. Methods: Here, we compared the transcriptome from the blood of subjects with diets supplemented with olives, nuts, or long-chain omega-3 fatty acids and identified the genes differentially expressed. The dietary genes obtained were subjected to network analysis to determine the main pathways, as well as the transcription factors and microRNA interaction networks to elucidate their regulation. Finally, a gene-associated disease interaction network was performed. Results: We identified several genes whose expression is altered after the intake of components of the Mediterranean diets compared to controls. These genes were associated with infection and inflammation. Transcription factors and miRNAs were identified as potential regulators of the dietary genes. Interestingly, caspase 1 and sialophorin are differentially expressed in the opposite direction after the intake of supplements compared to Alzheimer’s disease patients. In addition, ten transcription factors were identified that regulated gene expression in supplemented diets, mild cognitive impairment, and Alzheimer’s disease. Conclusions: We identified genes whose expression is altered after the intake of the supplements as well as the transcription factors and miRNAs involved in their regulation. These genes are associated with schizophrenia, neoplasms, and rheumatic arthritis, suggesting that the Mediterranean diet may be beneficial in reducing these diseases. In addition, the results suggest that the Mediterranean diet may also be beneficial in reducing the risk of dementia.

## 1. Introduction

Dietary patterns are associated with different disease risks. Whereas the Western diet increases the risk of cardiovascular disease and some types of cancers, other diets have some beneficial effects on health. The Mediterranean diet is rich in the consumption of fruits, vegetables, olive oil, fish, and nuts. It is characterized by the intake of food rich in polyphenols, monounsaturated fatty acids, and polyunsaturated fatty acids. It has been proposed that some components of the Mediterranean diet are beneficial for the individual’s health. Based on observation as well as randomized controlled studies, the Mediterranean diet is proposed to reduce the risk of developing several diseases. A beneficial effect of the diet on cardiovascular risks has been observed (reviewed in [1]), along with a decrease in blood pressure [2,3,4]. In addition, the Mediterranean diet is proposed to be beneficial for patients with diabetes mellitus by improving glycogenic regulation in patients [1,5,6]. Furthermore, several observational studies found that the Mediterranean diet reduced the risk of developing several cancers, such as colorectal, breast, stomach, liver, and head and neck cancer [1,7]. Moreover, Mediterranean diets may improve an individual’s resilience to Parkinson’s disease, depression, and dementia [1].

The beneficial effects of some of the components of the Mediterranean diet have been investigated. The main source of fat in the diet is olive oil. Olive oil contains high levels of monounsaturated fatty acids, as well as other biologically active components such as polyphenol [8]. Many studies have shown the benefits of olive oil on cardiovascular risk factors [9,10] and the benefits for patients with type 2 diabetes [5,11]. Olive oil also helps control the levels of plasma lipids and glucose in patients with metabolic syndrome [12]. Additionally, olive oil may be protective for some forms of cancer [13,14,15]. It has been suggested that the health benefits of olive oil are related to its anti-hypertensive, anti-inflammatory, and antioxidant effects [11,16,17,18]. 

Nuts, another component of the Mediterranean diet, are good sources of monosaturated fatty acids, polyunsaturated fatty acids, and other nutrients such as fibers, vitamin E, and L-arginine. Walnuts, for example, are rich in in long-chain omega-3 fatty acids (omega-3). Numerous epidemiologic studies have illuminated the beneficial impact of nut consumption on health outcomes (reviewed in [19]). Indeed, the consumption of nuts has been shown to have a positive effect on patients suffering from obesity, hypertension, diabetes mellitus, and cardiovascular diseases [20,21,22,23,24]. Nuts can reduce oxidative stress, inflammation, and blood pressure while helping with glycemic control [25,26,27,28].

Finally, fish consumption, characteristically high in a Mediterranean diet, has been proposed to have potential health benefits. Omega-3 are essential fatty acids that are found in fish oils. These n-3 fatty acids are composed of two crucial components: polyunsaturated fatty acids eicosapentaenoic acid (EPA) and docosahexaenoic acid (DHA). Studies have highlighted the beneficial effect of fish consumption and omega-3 on cardiovascular diseases, stroke, atherosclerosis as well as insulin resistance [29,30]. One of the possible mechanisms proposed for the health benefits was a decrease in plasma bioactive lipid components involved in insulin resistance and inflammation [31,32].

Microarray and high-throughput technologies for gene expression are essential tools for identifying differential patterns of gene expression that are characteristic of environmental determinants of health. The use of peripheral blood mononuclear cells (PBMC) to determine the effect of diet has shown that food intake can modify the blood’s proteome, metabolome, and gene expression profile [33,34,35]. Because PBMC can be easily and repeatedly collected, compared to liver, muscles, and adipocyte tissue, they are used frequently to study the impact of diet [35], including that of olive oil, olive leaves, nuts, and omega-3 consumption [36,37,38,39,40]. 

In this study, we took an integrative network-based approach to identify the genes that may be responsible for the beneficial effects of Mediterranean diets. Previously, we used a similar approach to reveal some of the mechanistic pathways involved in the development of Parkinson’s diseases, Alzheimer’s disease, and other dementias [41,42,43,44]. Among the dietary genes identified, we observed that the Nuclear Factor Interleukin 3 Regulated (*NFIL3*) was downregulated after the intake of all three supplements. In addition, Interleukin 8 (*IL8*), the Serine/Threonine Kinase 17b (*STK17B*), and Serpin Family B Member 2 (*SERPINB2*), and the Regulator of G Protein Signaling 1 (*RGS1*) were downregulated after two supplements were included in the diet. Pathway analysis determined that these genes were associated with infection and inflammation, suggesting potential health benefits of the Mediterranean diet in modulating the immune system. Gene-transcription factor and gene-miRNA network analyses identified important factors involved in the expression of dietary genes. Interestingly, we observed some shared transcription factors involved in the regulation of supplemented diets and dementia, indicating that the supplements may also be beneficial in reducing the risk of Alzheimer’s disease.

## 2. Materials and Methods

### 2.1. Analysis of PBMC Transcriptomic Studies

We used the curated database BaseSpace Correlation Engine (BSCE, Illumina, Inc., San Diego, CA, USA) to search for gene expression studies in different human diets [45]. Using the search terms “Mediterranean diet”, “olive diet”, “nut diet”, “fish oil”, “EPA”, “DHA”. “blood”, “human”, “RNA”, and “microarray”, we identified several studies with PMBC from people under different diets. Only human microarray studies with 5 samples or more for cases and controls and curated in BSCE were considered for analysis. In addition, analysis from PBMC collected from obese patients was not included in this analysis. Six microarrays met our inclusion criteria as of 1 October 2019. A description of microarray datasets included in this study is provided in Section 3.1. The arrays from olive oil and olive leaves diets were defined as “olive diet”, whereas the arrays with EPA/DHA were defined as “omega-3 diet”.

The differentially expressed genes were curated by BSCE. Statistical analyses were performed on log scale data. In the parametric test, variances were not assumed equal (Welch *t*-test). A *p*-value cutoff of 0.05 and a fold-change of 1.2 was applied to generate the final list of genes. Genes whose mean normalized test and control intensities are both less than the 20th percentile of the combined normalized signal intensities were removed. Final gene expression data from microarray studies were downloaded from BSCE (Table S1). A Venn diagram analysis was performed with the genes up or downregulated in the three olive diet arrays and the four fish oil arrays independently. The transcription factors Venn diagram was created using the website http://bioinformatics.psb.ugent.be/webtools/Venn/. Only genes that were differentially expressed in at least two olive diet arrays and at least two fish oil studies were included for further analysis. The list of genes can be found in Table S2.

### 2.2. Pathway Enrichment Analysis

Official gene symbols for the genes identified in the supplemented diets gene expression comparison were imported into NetworkAnalyst for pathway analyses (https://www.networkanalyst.ca/NetworkAnalyst/faces/home.xhtml) for pathway analyses using the Kyoto Encyclopedia of Genes and Genome (KEGG) pathway database [46]. NetworkAnalyst uses an enrichment network OverRepresentation Analyses (ORA). ORA is a statistical technique to identify gene sets or pathways that have a significant overlap with the selected genes of interest. In NetworkAnalyst, hypergeometric tests are used to compute the *p*-values [47,48].

### 2.3. Gene-Transcription Factors Interaction Analysis

Gene-transcription factors interactome was performed in NetworkAnalyst. Transcription factor and gene target data were derived from the Encyclopedia of DNA Elements (ENCODE) ChIP-seq data, ChIP Enrichment Analysis (ChEA), or JASPAR database [49,50,51]. ENCODE uses the BETA Minus Algorithm in which only a peak intensity signal <500 and the predicted regulatory potential score <1 is used. ChEA transcription factor targets the database inferred from integrating literature curated Chip-X data. JASPAR is an open-access database of curated, non-redundant transcription factor (TF)-binding profiles. A Venn diagram analysis was performed with the transcription factors identified with each database. Transcription factors were ranked according to network topology measurements including degree and betweenness centrality.

### 2.4. Gene-miRNA Interaction Analysis

The gene-miRNA interactome was performed in NetworkAnalyst. The Gene-miRNA Interactome was carried out from comprehensive experimentally validated miRNA-gene interaction data collected from TarBase and miRTarBase [52,53,54].

### 2.5. Gene-Disease Association Analysis

Gene-disease association analysis was performed in NetworkAnalyst. The literature curated gene-disease association information was collected from the DisGeNET database, a publicly available collection of genes and variants associated with human diseases [55]. 

### 2.6. Diet and Dementia Analysis

Our previous gene expression analysis identified 91 mild cognitive impairment (MCI) genes dysregulated in in the 2 arrays analyzed and 387 Alzheimer’s disease (AD) genes dysregulated in at least 2 out of the 4 arrays analyzed [42]. A Venn diagram analysis was carried out between the dementia genes previously identified and the genes identified in the present diet study using the InteractiVenn website (http://www.interactivenn.net/).

## 3. Results

### 3.1. Gene Expression Comparison of the Diet Supplements

**Figure 1:**
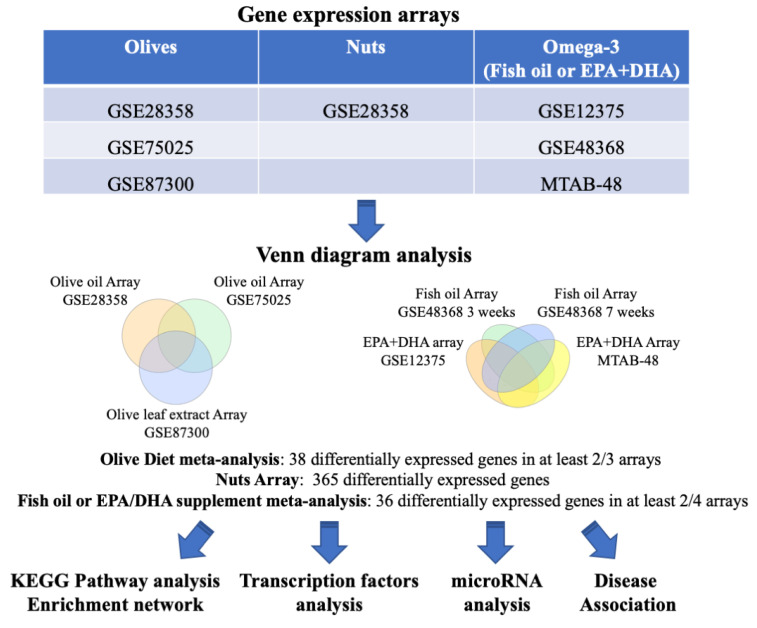
Flowchart of the study. The BaseSpace Correlation Engine (BSCE) was searched to identify microarray data from appropriate diet studies. Venn diagram analysis was used to identify shared differentially regulated genes. The genes shared by olive-, nuts- or omega-3-supplemented diets were analyzed for shared functional pathways, transcription factors, miRNAs regulation, and disease associations.

We first identified common genes differentially expressed in several diet studies. The datasets, platforms, and test samples for each study are listed in Table 1. The overall strategy of the study is presented in Figure 1. 

**Figure 2:**
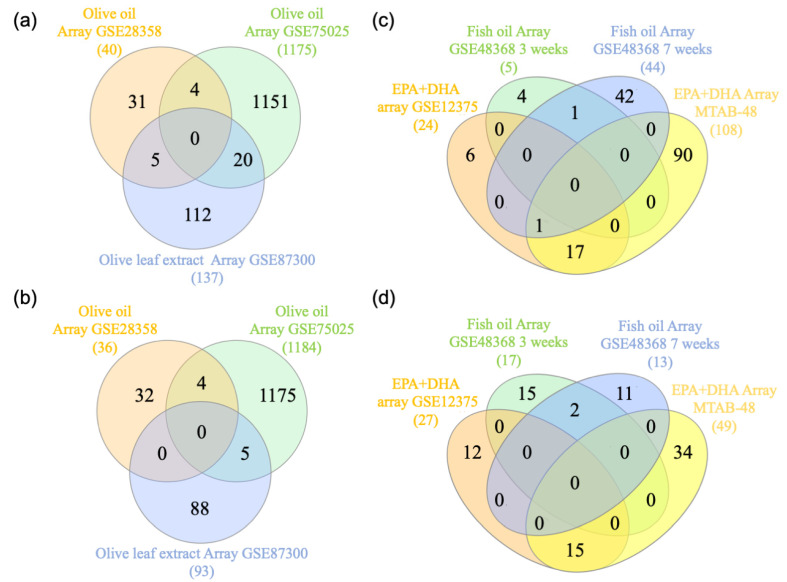
Venn diagram analysis of the genes up and downregulated in olive- and omega-3-supplemented diets. (**a**) and (**b**)**.** Genes in olive-supplemented diets: The genes downregulated (**a**) and upregulated (**b**) in the olive oil or leaf extracts arrays were downloaded from BSCE and used to create a Venn diagram using the following website http://www.interactivenn.net/. (**c**) and (**d**). Genes in Omega-3-supplemented diets: The genes downregulated (**c**) and upregulated (**d**) in the fish oil or eicosapentaenoic acid (EPA)/ docosahexaenoic acid (DHA) studies were downloaded from BSCE and used to create the Venn diagrams.

Three PBMC microarray datasets from olive-supplemented diets (olive oil or olive leaves) were identified in the BaseSpace Correlation Engine (BSCE) (GSE28358, GSE75025, and GSE87300). We used Venn diagram analysis to identify genes shared among the datasets from olive-based diets (Figure 2a,b, Table S1). A total of 38 genes were differentially expressed in at least two out of the three olive-supplemented diet arrays (Table S2). Similarly, four PBMC microarrays from omega-3-supplemented diet datasets (fish oil or DHA + EPA supplements) were obtained from BSCE and analyzed by (GSE48368, GSE48368, GSE12375, and E-MTAB-48) (Figure 2c,d, Table S1). A total of 36 differentially expressed genes were shared in at least two out of the four datasets analyzed (Table S2). Only one microarray dataset from a diet rich in nuts was available (GSE28358) and 365 genes were identified as differentially regulated (Table S2).

Next, we compared the genes differentially expressed in the diets. Interestingly, we found that the Nuclear Factor Interleukin 3 Regulated (*NFIL3*) was downregulated in the three types of diets. In addition, we observed that Interleukin 8 (*IL8*), the Serine/Threonine Kinase 17b (*STK17B*), and Serpin Family B Member 2 (*SERPINB2*) were downregulated in diets rich in olive and nuts. Additionally, the Regulator of G Protein Signaling 1 (*RGS1*) was downregulated in diets rich in nuts and omega-3. We did not find any upregulated genes shared between the diets.

### 3.2. Pathway Enrichment Analysis

**Figure 3:**
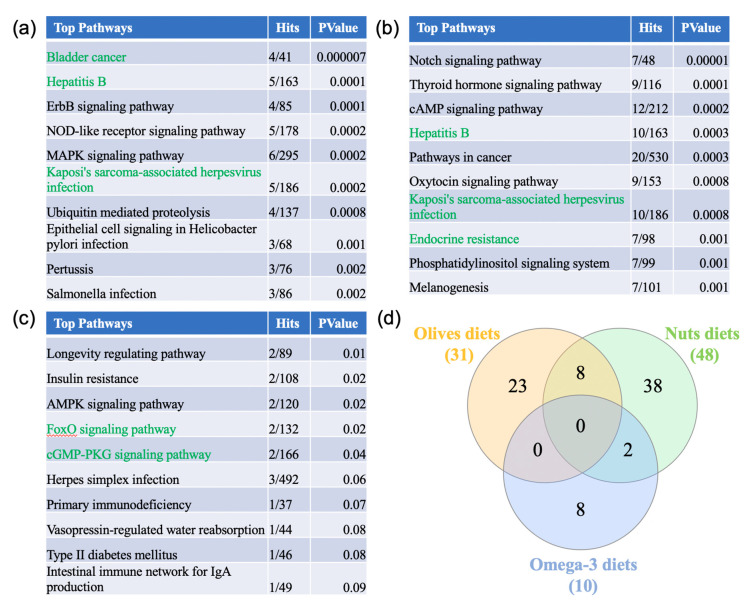
Pathway analysis. (**a**–**c**) The genes differentially expressed in 2 out of 3 olive supplement arrays, in the nuts supplement array, in at least 2 out of the 4 arrays from fish or EPA + DHA diets were obtained using Venn analysis. The gene lists were uploaded to https://www.networkanalyst.ca/NetworkAnalyst/faces/home.xhtml, where a list enrichment network analysis was performed using the KEGG database. The 10 tops pathways identified from the olive-, nuts-, or omega-3-supplemented diets are listed in (**a**), (**b**), and (**c**) respectively. (**d**) The pathways shared between the three types of Mediterranean diets were analyzed in a Venn diagram analysis.

A pathway analysis using NetworkAnalyst was performed to elucidate the functional and biological role of genes differentially expressed in the different diets. The pathway enrichment network analysis was performed using the Kyoto Encyclopedia of Genes and Genome (KEGG) database (Figure 3, Table S3). The 38 olive supplemented diet genes identified 31 pathways. The top 10 pathways were bladder cancer, Hepatitis B, ErbB signaling, NOD-like receptor signaling, MAPK signaling, Kaposi’s sarcoma-associated herpesvirus infection, ubiquitin-mediated proteolysis, epithelial cell signaling in *Helicobacter pylori* infection, pertussis, and salmonella infection. 48 pathways were identified from the genes differentiated regulated during a diet rich in nuts. The top 10 pathways were notch signaling, thyroid hormone signaling, hepatitis B, pathways in cancer, oxytocin signaling, Kaposi’s sarcoma-associated herpesvirus infection, endocrine resistance, phosphatidylinositol signaling, and melanogenesis. Finally, nine pathways were identified from the 36 differentially expressed genes in omega-3 rich diets. These pathways are longevity regulating, insulin resistance, 5′ AMP-activated protein kinase (AMPK) signaling, forkhead transcription factor family (FoxO) signaling, cyclic guanosine monophosphate-protein kinase G (cGMP-PKG) signaling, herpes simplex infection, primary immunodeficiency, vasopressin-regulated water reabsorption, type II diabetes mellitus, and intestinal immune network for IgA production.

Venn analysis showed that no pathway was shared by all three supplements. Moreover, we did not observe any pathways shared between the olive-based and omega-3-based diets. However, eight pathways were shared between the olive-based and nut-rich diets. These pathways were bladder cancer, Hepatitis B, Kaposi’s sarcoma-associated herpesvirus infection, Influenza A, proteoglycans in cancer, HTLV-I infection, endocrine resistance, and Chagas disease. In addition, we observed that cGMP-PKG signaling and FoxO signaling pathways were shared between the nuts and omega-3 diets. Interestingly several of the pathways associated with the different diets are associated with infection and inflammation.

### 3.3. Gene-Transcription Factors Interaction Analysis

**Figure 4:**
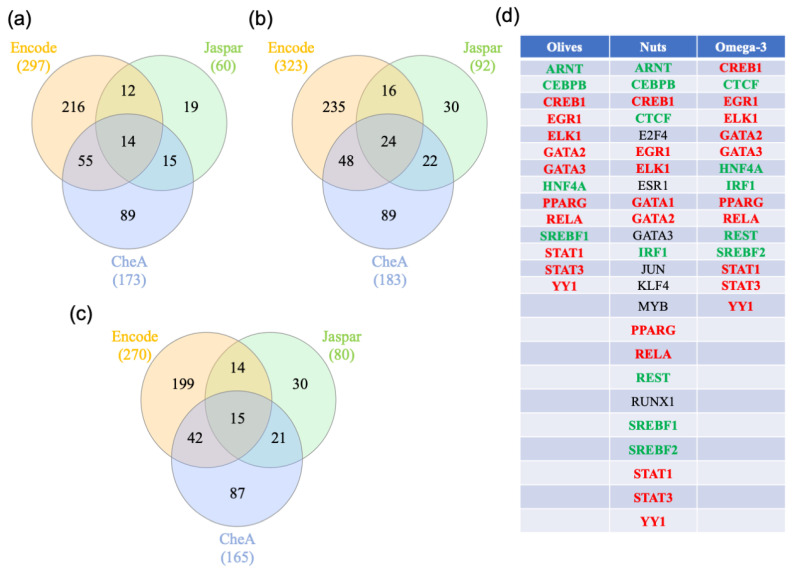
Transcription factor analysis. (**a**–**c**) Transcription factor analysis for each diet. The gene lists were uploaded to https://www.networkanalyst.ca/NetworkAnalyst/faces/home.xhtml. The gene–transcription factor interaction network was performed with ENCODE, ChEA, and JASPAR, and (**a**), (**b**), and (**c**) represent the results of the Venn analysis performed with olive-, nuts- and omega-3-genes, respectively. The transcription factors interacting with the supplement-regulated genes are listed in (**d**). Transcription factors in red are shared between the three supplements, the transcription factors in green are shared between two supplements, and the transcription factors in black are unique to a supplement.

To identify key regulators of the genes differentially expressed in the different types of diets, gene transcription factor interactomes were performed on NetworkAnalyst using three different databases (ENCODE, ChEA, and JASPAR) (Table S4). The transcription factors that were shared by all the databases were identified by Venn analysis (Figure 4a–c). The analysis identified 14, 24, and 15 transcription factors in the olive-, nuts-, and omega-3-supplemented diets, respectively. The list of these transcription factors is presented in Figure 4d. A total of 10 transcription factors shared among the three types of diets included CREB1, EGR1, ELK1, GATA2, GATA3, PPARG, RELA, STAT1, STAT3, and YY1. Eight transcription factors were identified in at least two analyses. HNF4A was shared between olive- and omega-3-supplemented diets. CTCF, IRF1, REST, and SREBF2 were shared between the nuts- and omega-3-supplemented diets. Finally, ARNT, CEBPB, and SREBF1 were shared between the olive- and nuts-supplemented diets.

### 3.4. Gene-miRNA Interaction Analysis

**Figure 5:**
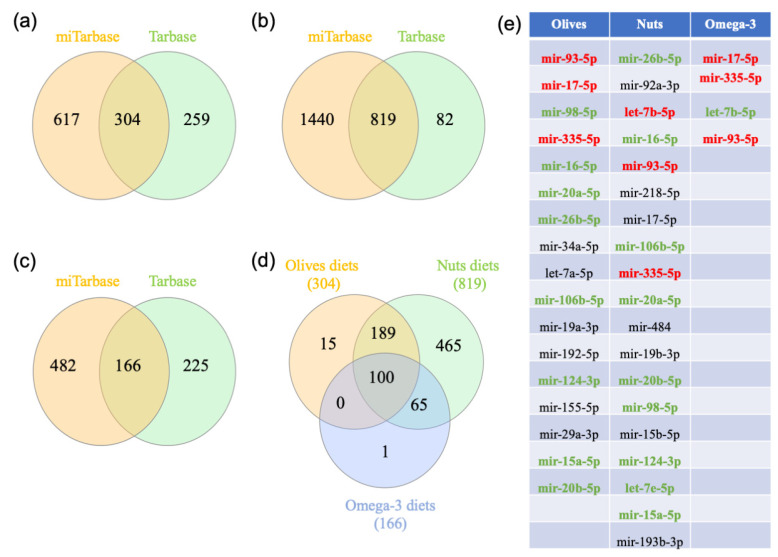
MicroRNA analysis. The diet gene lists were uploaded to https://www.networkanalyst.ca/NetworkAnalyst/faces/home.xhtml. A gene-microRNA interactome was performed using the TarBase and miRTarBase databases. (**a**), (**b**), and (**c**) represent the results of the Venn analysis performed with olive-, nuts- and omega-3-genes, respectively. (**d**) The miRNA shared between the three types of Mediterranean diets were analyzed in a Venn diagram analysis. The most significant miRNAs are listed in (**e**). MiRNAs in red are shared between the 3 supplements, the miRNAs in green are shared between 2 supplements, and the miRNAs in black are unique to a supplement.

To further understand the regulation of the expression of genes differentially expressed during diet supplementation, a gene-miRNA interaction network analysis was performed in NetworkAnalyst. Comprehensive experimentally validated miRNA-gene interaction data were collected from TarBase and miRTarBase. 304, 819, and 166 miRNAs were identified from an olive-rich diet, a nuts-supplemented diet, and an omega-3-supplemented diet, respectively (Figure 5 and Table S5). Interestingly, 100 miRNAs were shared in the three types of supplemented diets (Figure 5). In order to determine the most important miRNA, we performed the Venn analysis after selecting the top miRNAs using a degree cut off of five for the olive-rich and omega-3-supplemented diets and a degree cut off of 25 for the nuts-supplemented diet. This allowed us to identify 17, 19, and 4 miRNAs from an olive-rich diet, a nuts-supplemented diet, and an omega-3-supplemented diet, respectively (Table S5). Interestingly, three miRNAs were shared between the three diets (mir-93-5p, mir-17-5p, and mir-335-5p).

### 3.5. Gene-Disease Association Analysis

**Figure 6:**
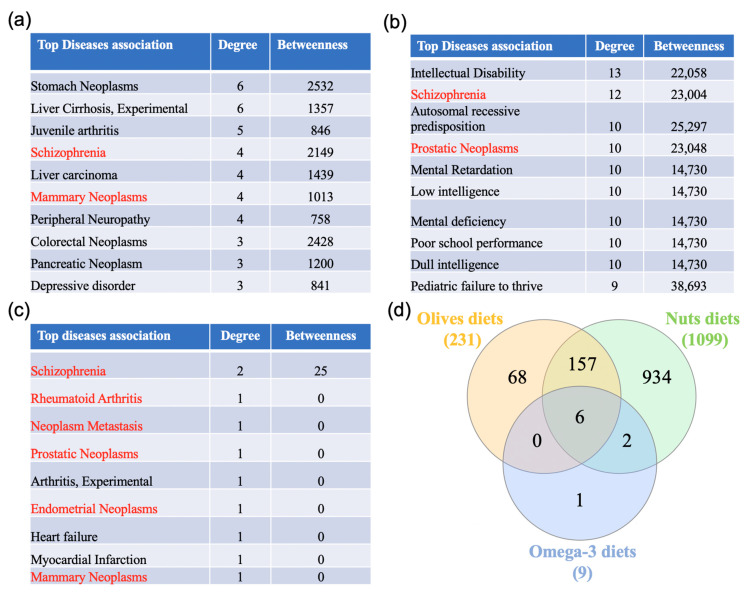
Disease association analysis. (**a**–**c**) The diet genes lists were uploaded to https://www.networkanalyst.ca/NetworkAnalyst/faces/home.xhtml to perform the disease association analysis. The diseases were ranked by decreasing degree followed by decreasing betweenness, and the top 10 diseases obtained from the olive-, nuts, and omega-2-supplemented diets are presented in (**a**), (**b**), and (**c**), respectively. (**d**) A Venn analysis was performed to determine the diseases shared between the different types of Mediterranean diets. The associated diseases in red are shared between the three supplements.

A gene-disease association network analysis was performed in NetworkAnalyst. The differentially expressed genes in the olive-, nuts- or omega-3-rich diets allowed for the identification of 231, 1099, and 9 associated diseases, respectively. These diseases were ranked by decreasing degree followed by decreasing betweenness (Figure 6 and Table S6). Interestingly, six associated diseases were shared between the three Mediterranean diet components: schizophrenia, mammary neoplasms, prostatic neoplasms, neoplasm metastasis, endometrial neoplasms, and rheumatoid arthritis. 

### 3.6. Diets and Dementia Analysis

In our previous study, we performed a gene expression comparison of publicly available arrays from blood samples obtained from mild cognitive impairment (MCI) and Alzheimer’s disease (AD) patients [42]. We obtained 91 MCI genes dysregulated in the two arrays analyzed and 387 AD genes dysregulated in at least two out of the four arrays analyzed. Interestingly, unhealthy eating habits might increase the risk to develop dementia [56] and numerous studies have highlighted the potential beneficial impact of the Mediterranean diet on cognition (reviewed in [57,58]). For example, nuts consumption has been shown to delay cognitive decline in aging populations and improves AD pathology [59,60]. Recently, the impact of a Mediterranean diet and the microbiota on neurodegeneration has been reviewed [61]. 

**Figure 7:**
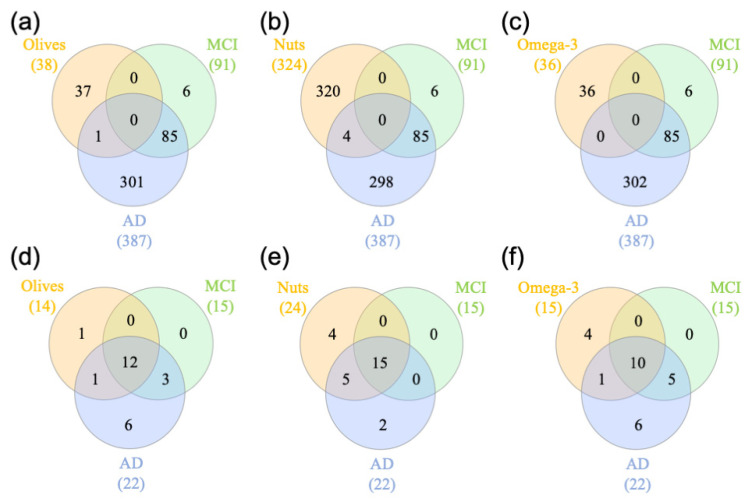
Comparison of gene expression data from supplemented diets to mild cognitive impairment (MCI) and Alzheimer’s disease (AD). (**a**–**c**) The shared genes between a specific supplemented diet, MCI, and AD were analyzed by Venn (**a**): olive-supplemented diet, (**b**): nuts-supplemented diet, (**c**): omega-3-supplemented diet. (**d**–**f**) The shared transcription factors between a specific diet, MCI, and AD were analyzed by Venn (**d**): Olive-supplemented diet, (**e**): Nuts-supplemented diet, (**f**): omega-3-supplemented diet.

We compared the genes dysregulated in MCI and AD patients to the genes identified in this study that are differentially expressed after diet supplementation [42]. Caspase 1 (*CASP1)* was shared between a diet supplemented with olives and AD patients (Figure 7a). In addition, four genes were shared between the diet supplemented with nuts and AD patients, including Actin-Related Protein 2/3 Complex Subunit 3 (*ARPC3)*, Sialophorin *(SPN)*, Neurobeachin Like 2 (*NBEAL2)*, and Mixed-Lineage Leukemia (*MLL*) (Figure 7b). No genes were shared between a diet rich in omega-3 and MCI and AD patients (Figure 7c). Interestingly, *CASP1* and *SPN* are regulated in dementia and the supplemented diets in the opposite direction, indicating that the supplements may be beneficial in reducing the risk of dementia. We observed that *CASP1* expression was upregulated in the olive-supplemented diet, whereas it was downregulated in the blood of AD patients. On the other hand, the expression of Sialophorin (*SLN*), also known as Leukosialin and CD43, was downregulated in a nut-rich diet, whereas it is upregulated in AD. In the blood, SLN is expressed at the surface of T cells and regulates multiple T cell functions. In the brain, SLN is expressed on the surface of microglia. Whereas our study indicated an upregulation in blood from AD patients, SLN protein expression was downregulated at the surface of microglial cells [62]. We also performed a pathways enrichment analysis between the genes identified in the supplemented diets and the genes identified in our previous dementia analysis. We did not find any shared pathways. 

Next, we compared the transcription factors obtained in the different diets to the previously identified transcription factors involved in MCI and AD regulation [42]. We observed a significant overlap between the diet and dementia analysis (Figure 7). A total of 12 transcription factors identified in the olive-supplemented diet were also identified in the dementia analysis (PPARG, EGR1, CREB1, ELK1, YY1, GATA2, GATA3, STAT1, CEBPB, RELA, STAT3, and SREBF1) (Figure 7d). A total of 15 transcription factors were shared between a nuts-supplemented diet and dementia (ELK1, EGR1, PPARG, YY1, STAT1, CREB1, GATA2, GATA3, CEBPB, RELA, STAT3, SREBF1, E2F4, JUN, and RUNX1) (Figure 7e). Finally, 10 transcription factors were regulating both the omega-3-supplemented diet and dementia genes (PPARG, YY1, ELK1, CREB1, GATA2, GATA3, EGR1, RELA, STAT1, and STAT3) (Figure 7f). Together, we identified 10 transcription factors that are involved in the regulation of the three different types of supplemented diets, MCI and AD (PPARG, EGR1, CREB1, ELK1, YY1, GATA2, GATA3, STAT1, RELA, and STAT3).

## 4. Discussion

### 4.1. Regulation of Gene Expression by Components of a Mediterranean Diet

In this study, we identified several differentially expressed genes that are shared between the various diets. The main finding is that the transcriptional repressor *NFIL3*, also known as E4 binding protein 4 E4BP4, was downregulated in all three supplemented Mediterranean diets. *NFIL3* can regulate the expression of several cytokines and is involved in the development of immune cells [63]. In addition, *NFIL3* is an important regulator of the circadian clock, which acts by repressing the expression of PER1 and PER2 [64]. Interestingly, the expression of *NFIL3* is regulated by nutrients. Indeed, the expression of *NFIL3* is activated by insulin and feeding, whereas fasting decreases its expression [65]. *NFIL3* can repress the expression of *FGF21*, a protein with anti-diabetic, and triglyceride-lowering properties [65]. In intestinal epithelial cells, *NFIL3* has been shown to regulate lipid storage and body composition in mice [66]. *NFIL3* also regulates the signaling processes involved in heart functions [67], and, in the brain, *NFIL3* blocks neuronal regeneration by competing with the transcription factor CREB [68]. Decreasing expression of *NFIL3* using siRNAs induced neurite outgrowth in a rat neuronal model [68].

*IL8* is downregulated in diets supplemented with olive oil and nuts. IL8 is a key mediator of inflammation. This cytokine functions as a chemoattractant allowing target cells, such as neutrophils, to migrate towards a site of infection. Interestingly, IL8 is one of the immunological signatures of excess body weight [69,70]. IL8 might play a role in some obesity-related metabolic complications [69]. Adherence to a Mediterranean diet was shown to reduce the plasma levels of IL8 and to delay atheroma plaque development [71]. Wine, consumed in moderate quantity, could also decrease the level of IL8. Different varieties of grapes, as well as their phenolic compounds, have been shown to reduce IL8 levels [72]. 

The serine-threonine kinase *STK17B*, also known as Death-Associated Protein Kinase-Related 2 (*DRAK2*), is downregulated in both diets rich in olive and nuts. Free fatty acid (FFA) increases the expression of STK17B, which participates in the apoptosis of islet β-cells [73]. STK17B has been proposed as a novel target in the treatment of diabetes. Indeed, knockdown of STK17B by siRNA attenuated FFA-induced islet β cells apoptosis [73]. In addition, STK17B upregulation was observed by pro-inflammatory cytokines such as IL-1β, and TNF-α. The downregulation of STK17B was able to prevent the inflammatory-induced cell death of insulinoma cells [74]. The chemical inhibition of STK17B has been proposed as a potential diabetes treatment [75]. Together, these studies suggested that the downregulation of STK17B could be involved in some of the health benefits of the Mediterranean diet.

*SERPINB2*, also known as plasminogen Activator Inhibitor 2 (PAI2), is downregulated in both diets rich in olive and nuts. The coagulation factor SERPINB2 inactivates the tissue plasminogen activator and urokinase. The role of SERPINB2 in diets is largely unknown. It has been shown that, in mice fed a high-fat diet, SERPINB2 promotes adipose tissue development [76]. Moreover, it has been shown that SERPINB2 expression is decreased in a methionine-supplemented diet [77].

*RGS1*, a regulator of the G-protein superfamily, is downregulated in both diets rich in nuts and omega-3. Polymorphic variants in *RGS1* have been linked to chronic inflammatory diseases such as celiac disease, multiple sclerosis, and type I diabetes [78,79,80]. RGS1 was upregulated in the epididymal white adipose tissue of high-fat diet-fed mice [81]. In addition, RGS1 expression was upregulated in both obese adipose tissue and atherosclerotic aortae models [82]. RGS1 was upregulated in both PBMC and brain samples from Alzheimer’s disease patients [83]. Altogether, whereas RGS1 is upregulated in different diseases, its expression is decreased with Mediterranean diets. 

### 4.2. Gene Expression Regulation by Transcription Factors and miRNA in Mediterranean Diets

Performing a gene-transcription factor interaction network, we identified 10 transcription factors that might regulate the genes differentially expressed in Mediterranean diets. The transcription factor CREB1 is increased by listroside, a purified olive secoirodoid derivative [84]. It has been proposed that listroside could ameliorate the mitochondrial function in an Alzheimer’s disease cell model [84]. In addition, CREB1 is proposed as an early biomarker candidate for obesity-induced pathophysiological changes in the colon [85]. PPARG is considered a key regulator of lipid metabolism [86]. It has been shown that a polymorphism in PPARG was associated with the effect of diet on the health of the individual [87]. Hydroxytyrosol, a component of olive oil, was shown to downregulate STAT3 in K562 cells [88]. Polyphenol extract from olive oil has also been shown to inhibit STAT3 in a model of rheumatoid arthritis [89]. Polymorphism in the *STAT3* gene might interact with higher saturated fat intake and increases the risk of abdominal obesity [90]. 

MicroRNAs are crucial in the regulation of gene expression and have been implicated in several diseases including metabolic disorders and insulin resistance (reviewed in [91]). We also performed a network analysis to reveal the microRNAs involved in the regulation of the differentially expressed genes by the supplemented diets and observed three shared microRNAs (miR-17-5p, miR-355-5p, and miR-93-5p). MiR-17-5p is involved in angiogenesis, proliferation, apoptosis, and autophagy [92,93], and it plays a role in several cancers, including hepatocellular carcinoma, osteosarcoma, leukemia, lung, gastric, colorectal, prostate, and breast cancer [93]. MiR-17-5p levels increase after high-fat diet consumption and activate adipogenic differentiation [94]. Interestingly, the plant polyphenol curcumin decreases miR-17-5p levels and inhibits adipogenesis [94]. However, another study on retinal inflammation indicated that high-fat diets for 8 weeks induced obesity and insulin resistance, as well as, decreased miR-17-5p [95]. Endoplasmic reticulum stress was proposed to trigger the reduction of miR-17-5p [95]. MiR17-5p reduces inflammation and lipid accumulation in an atherosclerosis model [96]. Finally, a role for miR-17-5p in aging has been proposed [97]. Surprisingly, whereas several miRNAs are decreased in aging brains, an increase in miR-17-5p levels was observed [98]. However, in neurodegenerative diseases such as Alzheimer’s disease, miR-17-5p expression is inhibited and this may be responsible for an increase in APP protein levels [99]. Flavonoids are components of Mediterranean diets that inhibit oxidative stress and neuroinflammation. MiR-355-5p was one of the miRNAs potentially regulated after exposure to flavonoids [100]. In a dietary methionine restriction mouse model, miR-355-5p was elevated in the bone marrow and might be involved in osteoblast differentiation and function [101]. Finally, an increase of miR-355-5p was observed in white adipose tissue of ob/ob mice and mice on a high-fat diet [102]. Altogether, these studies indicate that miR-355-5p might be involved in the network of genes differentially regulated by the Mediterranean diet and, consequently, in the cellular effect of this diet. Little is known about the impact of diets on miR93-5p; however, its expression increases in coronary artery disease patients, suggesting the potential use of this miRNA as a diagnostic marker [103]. 

### 4.3. Mediterranean Diets and Disease Association

Schizophrenia was identified as one of the six diseases associated with olive-, nuts-, and omega-3-rich diets. Schizophrenia patients have a shorter lifespan due to metabolic and cardiovascular disease and often practice unhealthy dietary habits [104,105,106]. Improving the diet of Schizophrenic patients could be very beneficial [107]. For example, eating a Mediterranean diet improved cardiovascular risks in Schizophrenic patients [108]. In addition, the omega-3 fatty acid supplement was beneficial for Schizophrenic patients by improving their psychopathology, reducing tardive dyskinesia, and attenuating the risk of conversion to psychosis in patients [109,110,111]. 

We also observed that rheumatoid arthritis is associated with all three types of supplements used in this study. Compared to a Western diet, eating a Mediterranean diet reduces inflammatory activity, improves physical function, and vitality [112]. In a rat model of rheumatoid arthritis, the gavage of hydroxytyrosol, a typical virgin olive oil phenolic compound, decreased both acute and chronic inflammation [113]. Rheumatoid arthritis patients consume less monounsaturated fatty acids than healthy individuals [114]. Monounsaturated fatty acids are a component of the Mediterranean diet, and, therefore, it may be beneficial for these patients [114]. Although several trials and systemic reviews show that a Mediterranean diet might improve the patient’s condition, further clinical studies are necessary to recommend the use of this diet as an adjunct therapy to standard treatment [115,116,117]. 

An inverse correlation between the consumption of a Mediterranean diet and cancer risk has been proposed for several neoplasms, including breast and prostate [118,119,120,121]. However, recent meta-analyses suggested that further investigations are needed for a better assessment [122,123]. Moreover, it is interesting to note that, in addition to possibly decreasing the risk of cancer development, the Mediterranean diet may improve the quality of life of a cancer survivor [124,125].

### 4.4. Limitations

This comparison of gene expression using diets with different supplements associated with Mediterranean diets is based on publicly available microarray data, and, therefore, several limitations should be considered. For example, the sample size could influence the power of the analysis. Moreover, the studies were performed at different sites, following different protocols, and these differences might also influence the results. The present study was performed using the statistical criteria described in the methods section with the datasets that were available at the time of the analysis. The gene expression data were curated using the database BSCE. The differential expression of genes at the lower end of the thresholds was applied (1.2 FC, *p* < 0.05) in order to produce a larger set of genes for the initial analysis. These criteria may produce a higher likelihood of false positives. Alternatively, future studies could use more stringent thresholds initially to reduce the number of false positives. It is also important to note that datasets are updated routinely and thus analysis of the data with newer datasets may yield different results. In addition, the results may be influenced by the participant themselves. Their genetics, age, sex and disease risk status, as well as their lifestyle, can influence gene expression. To improve the power of this gene expression comparison, we had to combine data from individuals who had different characteristics (Table 1). Future studies with many more participants will be needed to determine if the Mediterranean diet influences gene regulation differently in young and aging populations, males and females, and in healthy participants and those with a high risk of cardiovascular disease. In addition, it would be interesting to analyze the effect of Mediterranean diet on populations of individuals with different health conditions such as cancer, dementia, or metabolic disorders to determine if there is a benefit. Finally, whereas the individual component of the Mediterranean diet was used for this study, it would be interesting to determine the effect of the full Mediterranean diet and to determine if a synergistic effect of the component would be beneficial.

## 5. Conclusions

Comparing gene expression profiles, we determined that *NFIL3*, *IL8*, *STK17B*, *SERPINB2*, and *RGS* were differentially expressed by supplementation of different components of the Mediterranean diets. These genes were associated with infection and inflammation. The Mediterranean differentiated genes were regulated by several key transcription factors (HNF4A, IRF1, REST, CTCF, and SREBF2) and miRNA (miR-17-5p, miR-335-5p, miR-93-5p). Finally, we determined several diseases for which the Mediterranean diet could be beneficial (schizophrenia, several neoplasms, rheumatoid arthritis, and dementia).

## Figures and Tables

**Table 1:** Gene expression datasets used in this study. The peripheral blood mononuclear cells (PBMC) transcriptomic studies that were selected for analysis are presented in the table. Age is indicated in years as mean ±SD, or mean alone, or mean (range) depending of the information available. EPA+DHA: eicosapentaenoic acid and docosahexaenoic acid.

Diets	Datasets	Platform	Sample #	Age	Sex (%F)	Health	Reference
Mediterranean diet + Olive oil	GSE28358	GPL571 [HG-U133A_2] Affymetrix Human Genome U133A 2.0 Array	12	62 ± 8	45	High risk of coronary artery disease	[36]
Olive oil	GSE75025	GPL10558 Illumina HumanHT-12 V4.0 expression beadchip	12	29 ± 2	50	Healthy	[37]
Olive leaf extract	GSE87300	GPL13667 [HG-U219] Affymetrix Human Genome U219 Array	15	32	0	Healthy	[38]
Mediterranean diet + Nuts	GSE28358	GPL571 [HG-U133A_2] Affymetrix Human Genome U133A 2.0 Array	10	63 ± 6	45	High risk of coronary artery disease	[36]
Fish oil 3 weeks	GSE48368	GPL10558 Illumina HumanHT-12 V4.0 expression beadchip	17	27.2 ± 6.9	71	Healthy	[39]
Fish oil 7 weeks	GSE48368	GPL10558 Illumina HumanHT-12 V4.0 expression beadchip	17	27.2 ± 6.9	71	Healthy	[39]
EPA + DHA	GSE12375	GPL7144 NuGO array (human) NuGO_Hs1a520180	23	69.9 (67-76)	35	Healthy	[40]
EPA + DHA	E-MTAB-48	A-AFFY-111—Affymetrix Custom Array	23	69.9 (67-76)	35	Healthy	[40]

## Supplementary Materials

The following are available online at https://www.mdpi.com/2072-6643/12/12/3765/s1, Table S1. Genes down and up-regulated in the selected arrays; Table S2. Genes shared in the different diets; Table S3. Enrichment pathways; Table S4. Gene-Transcription factor analysis; Table S5. miRNA analysis; Table S6. Disease association analysis.
